# Supplementary material for: Phenolic and Anthocyanin Compounds and Antioxidant Activity of Tamarillo (Solanum betaceum Cav.)
Source: Antioxidants (Basel). 2020 Feb 18;9(2):169. doi: 10.3390/antiox9020169 (PMC7070485; doi:10.3390/antiox9020169)
Supplement: Supplementary file 1 [file antioxidants-09-00169-s001.pdf]

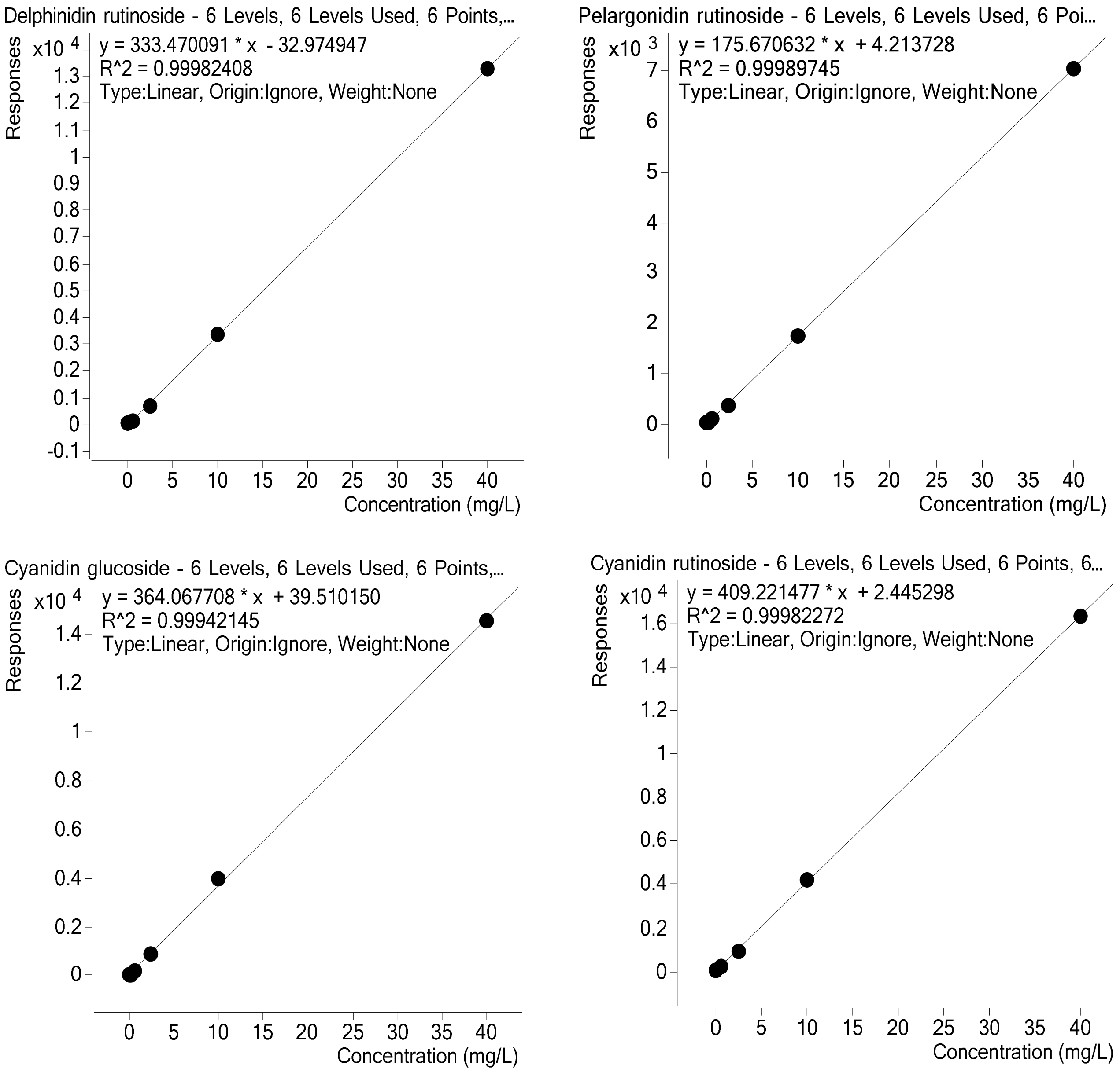

Figure S1. Standard curves of four anthocyanins detected in tamarillo using Agilent Chemstation Software (Agilent Technologies, Australia) for LC-MS

Table S1. Method validation for quantification of polyphenols and anthocyanins present in tamarillos.

Analytes	RT (min)	Precursor ion (m/z)	Product ion (m/z)	Collision energy (V)	Regression equation	Linear fit correlation coefficient²	Calibration range ($mg.L^{-1}$)	Limit of detection ($\mu g.L^{-1}$)	Limit of quantification ($\mu g.L^{-1}$)	Ion mode
Polyphenols										
Chlorogenic acid	8.908	353.0	191.0	13	y = 9149.52 x – 1284.97	0.9971	0.0977 – 6.25	0.1920	0.5817	Negative
Caffeic acid	9.381	179.0	135.0	13	y = 14247.18 x – 5425.88	0.9994	0.0879 – 11.25	0.0419	0.1270	Negative
p-coumaric acid	11.651	163.0	119.0	12	y = 2839.08 x + 114.77	0.9992	0.0586 – 7.5	0.0911	0.2761	Negative
Ferulic acid	12.827	193.0	134.0	12	y = 822.08 x + 37.54	0.9999	0.1074 – 13.75	0.1878	0.5692	Negative
Gallic acid	2.089	169.0	125.0	10	y = 37448.95 x + 1792.43	0.9964	0.0488 – 6.25	0.2980	0.9030	Negative
Ellagic acid	13.557	301.0	145.0	36	y = 787.13 x – 24.83	0.9992	0.0732 – 9.375	1.7155	5.1984	Negative
Kaempferol	17.228	285.0	239.0	24	y = 380.98 x – 80.54	0.9993	0.0586 – 7.5	0.8451	2.5610	Negative
Catechin	9.020	289.0	245.0	6	y = 7589.21 x – 1046.10	0.9985	0.4883 – 31.25	0.6137	1.8598	Negative
Epicatechin	10.870	289.0	245.0	8	y = 8305.81 x – 5475.92	0.9937	0.2441 – 15.625	0.7679	2.3270	Negative
Rutin	13.622	609.0	300.0	40	y = 23979.25 x – 1254.12	0.9992	0.0488 – 6.25	0.0092	0.0279	Negative
Kaempferol rutinoside	14.852	593.0	285.0	30	y = 10917.23 x – 340.32	0.9984	0.0488 – 6.25	0.0287	0.0869	Negative
Isorhamnetin rutinoside	15.096	623.0	315.0	28	y = 8272.36 x – 297.53	0.9991	0.0488 – 6.25	0.0408	0.1237	Negative
Anthocyanins										
Delphinidin rutinoside	4.890	609.1	300.1	33	y = 333.47x – 32.98	0.9998	0.0391 – 40.0	2.3044	6.9831	Negative
Cyanidin glucoside	5.064	447.1	284.1	21	y = 364.07x + 39.51	0.9994	0.0391 – 40.0	3.8123	11.5523	Negative
Cyanidin rutinoside	5.396	593.2	284.1	33	y = 409.22x + 2.45	0.9998	0.0391 – 40.0	1.8851	5.7124	Negative
Pelargonidin rutinoside	5.848	577.2	269.1	25	y = 175.67x + 4.21	0.9999	0.0391 – 40.0	3.3400	10.1212	Negative

RT: retention time (minute)

m/z: mass-to-charge ratio
